# Group 2 Innate Lymphoid Cells Exhibit Tissue-Specific Dynamic Behaviour During Type 2 Immune Responses

**DOI:** 10.3389/fimmu.2021.711907

**Published:** 2021-08-17

**Authors:** Laurence S. C. Lok, Jennifer A. Walker, Helen E. Jolin, Seth T. Scanlon, Masaru Ishii, Padraic G. Fallon, Andrew N. J. McKenzie, Menna R. Clatworthy

**Affiliations:** ^1^Molecular Immunity Unit, Department of Medicine, MRC Laboratory of Molecular Biology, University of Cambridge, Cambridge, United Kingdom; ^2^Cambridge Institute for Therapeutic Immunology and Infectious Diseases, University of Cambridge, Cambridge, United Kingdom; ^3^Department of Immunology and Cell Biology, Graduate School of Medicine, Osaka University, Osaka, Japan; ^4^Immunology Frontier Research Center, Osaka University, Osaka, Japan; ^5^Medical Research Council Laboratory of Molecular Biology, Cambridge, United Kingdom; ^6^School of Medicine, Trinity College Dublin, Dublin, Ireland; ^7^Cellular Genetics, Wellcome Sanger Institute, Hinxton, United Kingdom

**Keywords:** Innate lymphoid cell, lymph node, mucosa, type 2 inflammation, intravital imaging

## Abstract

Group 2 innate lymphoid cells (ILC2s) are early effectors of mucosal type 2 immunity, producing cytokines such as interleukin (IL)-13 to mediate responses to helminth infection and allergen-induced inflammation. ILC2s are also present in lymph nodes (LNs) and can express molecules required for antigen presentation, but to date there are limited data on their dynamic behaviour. We used a CD2/IL-13 dual fluorescent reporter mouse for *in vivo* imaging of ILC2s and Th2 T cells in real time following a type 2 priming helminth infection or egg injection. After helminth challenge, we found that ILC2s were the main source of IL-13 in lymphoid organs (Peyer’s patches and peripheral LNs), and were located in T cell areas. Intravital imaging demonstrated an increase in IL-13^+^ ILC2 size and movement following helminth infection, but reduced duration of interactions with T cells compared with those in homeostasis. In contrast, in the intestinal mucosa, we observed an increase in ILC2-T cell interactions post-infection, including some of prolonged duration, as well as increased IL-13^+^ ILC2 movement. These data suggest that ILC2 activation enhances cell motility, with the potential to increase the area of distribution of cytokines to optimise the early generation of type 2 responses. The prolonged ILC2 interactions with T cells within the intestinal mucosa are consistent with the conclusion that contact-based T cell activation may occur within inflamed tissues rather than lymphoid organs. Our findings have important implications for our understanding of the *in vivo* biology of ILC2s and the way in which these cells facilitate adaptive immune responses.

## Introduction

Group 2 innate lymphoid cells (ILC2s), a relatively recently characterised ILC subset (1–3), are effectors of type 2 immunity. In helminth infection and allergic inflammation, ILC2s are early responders to epithelium-derived tissue alarmins, interleukin (IL)-25, IL-33 and thymic stromal lymphopoietin (TSLP), producing type 2 cytokines including IL-5 and IL-13 (3–5). Indeed, following infection with *Nippostrongylus brasiliensis*, a helminth frequently used to study type 2 immunity, ILC2-derived IL-13 is critical for worm expulsion from the gut (3, 6). IL-13, signalling *via* STAT6, promotes worm expulsion by stimulating eosinophil recruitment and production of resistin-like molecule beta. This, in turn, affects worm nutrition, goblet cell mucus secretion and smooth muscle contraction (7, 8). Inflammatory ILC2s expand in the small intestine lamina propria and mobilise *via* lymphatics into the circulation, transiently seeding distant organs, including the lungs, to further promote pathogen defence (9, 10). Following *N. brasiliensis* infection, the main early source of IL-13 was shown to be from migratory inflammatory ILC2s not tissue resident ILC2s (11). However, another study showed using fate mapping that circulating ILC2s were of intestinal origin early post-infection, but of pulmonary origin late post-infection (12). Therefore inflammatory ILC2s may be a short-lived subset that migrates from tissues following infection to act as early source of type 2 cytokines. In contrast, ILC2s have been implicated in the pathogenesis of allergic diseases, particularly asthma, with increased ILC2 numbers observed in the airways of asthmatics (13, 14).

While type 2 cytokine secretion represents the predominant mechanism by which ILC2s influence other innate and adaptive immune cells (15, 16), they may also function as antigen-presenting cells. ILC2s express major histocompatibility complex II (MHCII), with higher proportions of ILC2s expressing MHCII in lymphoid than non-lymphoid tissues (17). *In vitro*, ILC2s can present antigen *via* MHCII and stimulate CD4 T cell activation and Th2 cytokine production. CD4 T cells in turn can stimulate ILC2 cytokine production *via* IL-2 (17, 18). *In vivo*, MHCII-deficient ILC2s showed impaired ability to mediate *N. brasiliensis* worm expulsion (17). ILC2s can also contribute to T cell activation by expressing co-stimulatory molecules, such as OX40 ligand which was shown to be required for the type 2 response against *N. brasiliensis* (19).

Secondary lymphoid organs, including lymph nodes (LNs), and small intestinal Peyer’s patches (PPs), are important structures for T cell activation and the generation of adaptive immune responses (20). ILC2s have been identified, by confocal microscopy, within interfollicular T cell areas of peripheral LNs (21) and in peripheral areas of small intestinal PPs (22). Intravital multiphoton microscopy is a powerful tool for the dynamic imaging of immune cells in lymph nodes and tissues (23–25), enabling interactions between antigen-presenting cells, including dendritic cells (DCs) and B cells, and CD4 T cells to be visualised in real time (26–28). These studies defined three sequential stages of T cell-DC interaction, with prolonged interactions of more than an hour observed in stage 2, around 12 hours after the initial encounter with antigen-loaded DCs, culminating in T cell activation and cytokine production (26). Notably, the duration of these stage 2 interactions between DCs and CD4 T cells varies according to context, with Th2-polarising conditions associated with shorter interactions compared with Th1-polarising conditions (29).

Specific *in vivo* visualisation of ILC2s using a single fluorescent reporter has been challenging, as they express canonical Th2 cytokines and transcription factors in common with Th2 cells, making them difficult to distinguish (30, 31). Intravital imaging of ear skin, in mixed bone marrow T cell-deficient CXCR6-green fluorescent protein (GFP) (*Rag1* ^-/-^ *Cxcr6* ^gfp/+^) and T cell-replete (*Rag1* ^+/+^mT^+^) chimeric mice, showed ILC2s moving at similar speed to migratory DCs and forming interactions with mast cells, although GFP^+^ cells were shown to be positive for CD90 (not ILC2-specific) (32). *Ex vivo* imaging of sectioned lung explants from IL-13-GFP reporter mice, with CD4 T cells labelled by *ex vivo* staining of tissue sections and GFP^+^ cells demonstrated to be CD4^neg^ by flow cytometry, showed IL-13-GFP ILC2s moving at higher speeds than CD4 T cells (33). To date, the dynamic behaviour of ILC2s within the small intestinal mucosa and within lymphoid tissues in the gut and periphery has not been examined, and interactions with CD4 T cells have not been directly visualised.

Here, we undertook intravital imaging on CD2-GFP/IL-13-tdTomato dual fluorescent reporter mice, in which ILC2s and Th2 cells were readily distinguishable, to interrogate the dynamic behaviour of IL-13^+^ ILC2s in skin- and gut-draining lymphoid organs and within the small intestinal mucosa, both in homeostasis and following helminth infection or egg challenge, quantifying IL-13^+^ ILC2 motility and duration of interactions with T cells.

## Materials and Methods

### Mice

CD2-green fluorescent protein (GFP) (34)/IL-13-tdTomato double reporter mice were used for imaging of ILC2s and T cells. Mice were bred and maintained in specific pathogen free conditions at the Laboratory of Molecular Biology, Cambridge, UK. All experimental procedures were approved in accordance with the Animals (Scientific Procedures) Act 1986 UK.

### Helminth Models

For *N. brasiliensis* infection, live stage 3 N*. brasiliensis* larvae were injected subcutaneously (400 larvae in 200 µl of phosphate-buffered saline (PBS) per mouse) on day 0. Intravital imaging of PP and small bowel was performed on day 5, an early time-point following larval migration into the gut (8). For *Schistosoma mansoni* immunisation, *S. mansoni* eggs were inactivated by ultraviolet irradiation and injected subcutaneously into left hind hock (5000 eggs in 25 µl of PBS per mouse), with PBS control injected into right hind hock on day 0. Intravital imaging of both popliteal LNs was performed on day 1.

### Antibodies and Flow Cytometry

Anti-mouse antibodies were purchased from BioLegend, eBioscience or Thermo Fisher for staining, including: CD3-BV510 (145-2C11), IL-7Rα-BV605 (A7R34), ICOS-PerCP.Cy5.5 (C398.4A), CD5-PE.Cy7 (53-7.3), CD8-PE.Cy7 (53-6.7), CD19-PE.Cy7 (eBio1D3), NK1.1-PE.Cy7 (PK136), CD11b-PE.Cy7 (M1/70), CD11c-PE.Cy7 (N418), Gr-1-PE.Cy7 (RB6-8C5), Fcϵ1-PE.Cy7 (MAR-1), TER-119-PE.Cy7 (TER-119), Siglec-F-Alexa Fluor 647 (E50-2440) and Live/Dead-A780. For flow cytometry, single-cell suspensions were generated and antibodies used at 1:200 for staining. Data were acquired using LSRFortessa flow cytometer and FACSDiva software, and analysed using FlowJo software.

### Confocal Microscopy

Tissues were fixed in 1% paraformaldehyde (PFA) overnight at 4°C, dehydrated in 30% sucrose and frozen in optimum cutting temperature (OCT) compound (VWR). Frozen sections were stained with antibodies (1:100) in buffer containing 0.1 M TRIS, 1% bovine serum albumin (Reagent diluent, R&D), 1% mouse serum and 0.3% Triton X-100 (Sigma) overnight at 4°C. Images were acquired using a Leica TCS SP8 microscope and 40X oil immersion objective.

### Intravital Imaging

Each mouse was anaesthetised using inhaled isoflurane and kept warm on an electric heat pad. Hair was removed by shaving and depilatory cream. For popliteal LN imaging, the mouse was placed prone on a custom-made stage, and the popliteal LN was surgically exposed under a dissecting microscope. For gut imaging, a small midline abdominal incision was made, the mouse placed laterally, and a loop of small bowel containing PP externalised. Following surgical preparation the mouse was transferred to the imaging box kept at 36°C throughout, and intravital imaging performed with a Leica TCS SP8 microscope and 25X water objective, with one Z stack every 40 seconds or less, and optical sections under 2 µm. Single-photon excitation lasers were used at 488 nm and 561 nm for GFP and tdTomato, respectively, and two-photon excitation (Chameleon Vision-S tuneable Ti : Sapphire multiphoton laser) with laser at 920 nm was used for second harmonic generation.

### Image Processing and Data Analysis

Intravital movies and confocal images were processed using Imaris (Bitplane) and ImageJ (NIH). Cells were tracked using either the ‘surfaces’ or ‘spots’ function and dynamic parameters such as mean speed generated. Track straightness is defined as the ratio of cell displacement over track length. Time-lapse movies were recorded at 10 frames per second (fps) or slower (as indicated) for clarity of presentation. Data were analysed using GraphPad Prism, with statistical analysis performed using one-way ANOVA with Holm-Sidak correction for between-group multiple comparisons. Data are shown as mean ± SEM unless otherwise indicated. *p* < 0.05 was considered statistically significant.

## Results

### ILC2s Are Present in PP at Baseline and Increase in Number Following Helminth Infection

In order to visualise ILC2s *in vivo* we used CD2-GFP/IL-13-tdTomato reporter mice, in which T cells were CD2^+^ (GFP^+^), Th2 cells CD2^+^IL-13^+^ (GFP^+^tdTomato^+^) and ILC2s CD2^neg^IL-13^+^ (tdTomato^+^), allowing ILC2s to be easily distinguished from Th2 cells, with CD2^+^IL-13^neg^ T cells acting as *in vivo* controls. Flow cytometric analysis of lymphoid tissues confirmed that IL-13^+^ cells were Lin^neg^Siglec-F^neg^CD2^neg^IL-7Rα^+^ICOS^+^ ILC2s (**Figures S1A, B**), which were negative for CD3 (**Figure S1C**). There was no IL-13 expression in Lin^+^ cells (**Figure S2A**). However, not all ILC2s were IL-13^+^ (**Figures S2A, B**), a potential limitation of this reporter.

**Figure 1:**
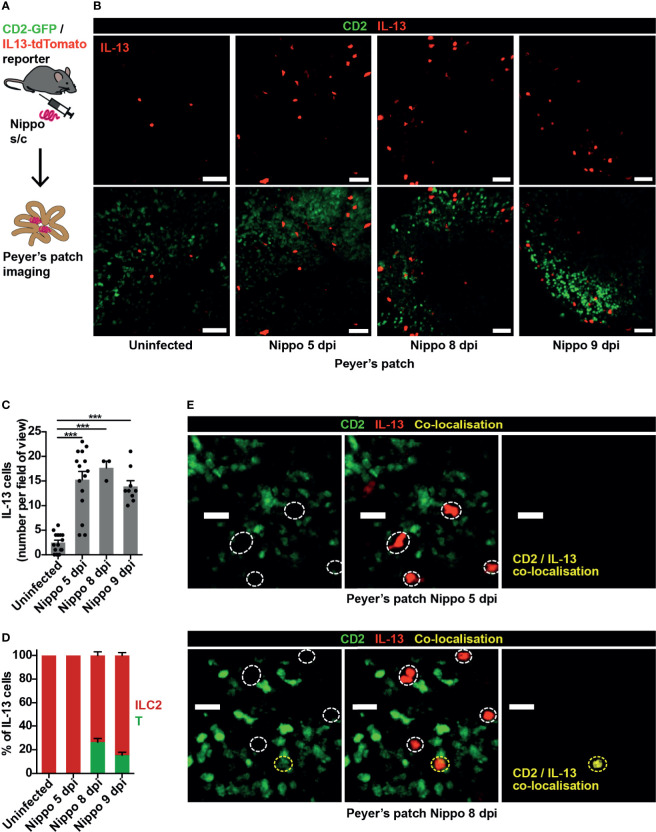
ILC2s are present in PP at baseline and expand following helminth infection. **(A)** Schematic of experimental setup. **(B)** Intravital images of IL-13^+^ ILC2s in PP in mice uninfected and 5, 8, 9 days post infection (dpi) with *N. brasiliensis* (Nippo); scale bar 50 µm, Z 40 µm. **(C, D)** Quantification of **(C)** ILC2 numbers and **(D)** proportions of IL-13^+^ T cells (CD2^+^) *vs* ILC2s (CD2^neg^) per field of view; ***p < 0.001 *vs* uninfected. Pooled data from 1-5 movies from 1-3 mice per time-point, each dot representing one field of view, total of 444 cells in 42 fields of view examined. **(E)** Intravital images of PP 5 and 8 dpi showing co-localisation of CD2 and IL-13; scale bar 20 µm, Z 50 µm.

The nematode *N. brasiliensis* is commonly used to model type 2 inflammation, with larvae entering through the skin and migrating to the lungs and small bowel causing an inflammatory response (8). We observed few IL-13^+^ ILC2s within small intestinal PP in homeostasis, but at 5 days post-infection (dpi) with *N. brasiliensis*, soon after worm arrival in the gut, ILC2 numbers in PP significantly increased. This persisted to 9 dpi, when worm expulsion is expected to occur (**Figures 1A–C**). At 5 dpi, Th2 cells (evidenced by co-localisation of GFP and tdTomato) were not observed, implicating ILC2s as the principal source of IL-13 in the PP at this early time-point. However, by day 8, around a quarter of the IL-13^+^ cells visualised in PP were T cells (**Figures 1D, E**).

### PP ILC2s Show Altered Dynamics Following Helminth Infection

**Figure 2:**
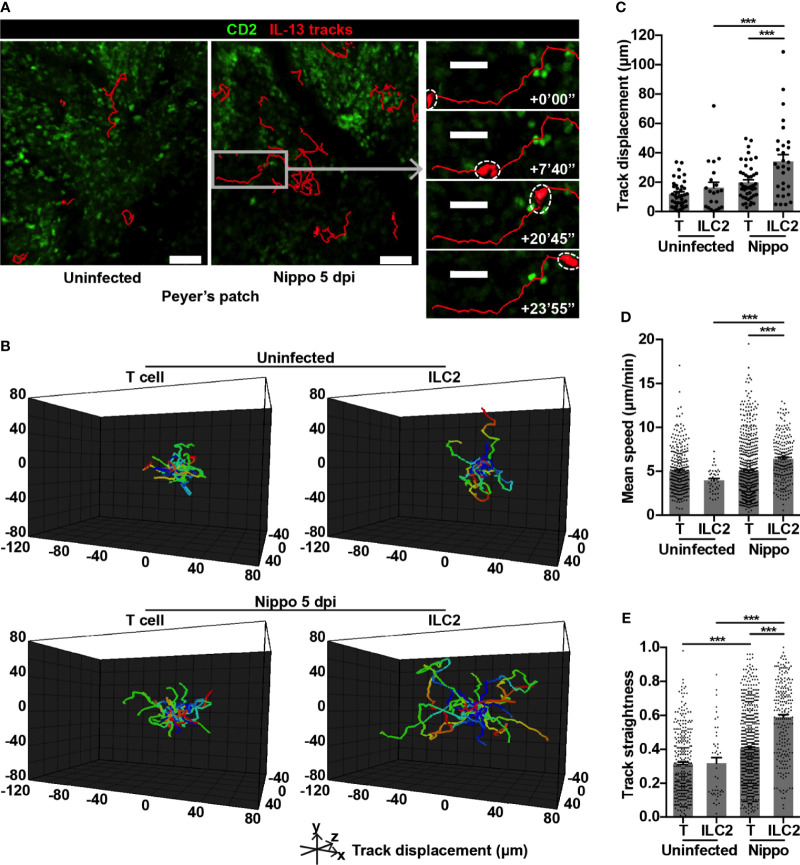
PP ILC2s show altered movement following helminth infection. **(A)** Intravital images of ILC2 cell tracks in PP uninfected and 5 dpi, with inset showing example of prolonged cell track 5 dpi; scale bar 50 µm (inset 30 µm), Z 48 µm. **(B)** Cell tracks from one uninfected movie and one 5 dpi movie each 45 minutes in duration, with starting positions centralised. **(C)** Quantification of cell track displacement from **(B)**; ***p < 0.001. **(D, E)** Quantification of **(D)** mean speed and **(E)** track straightness (directionality) of PP ILC2s and T cells; ***p < 0.001. Pooled data from 6-9 movies from 3 mice per condition, each dot representing one cell track.

**Figure 3:**
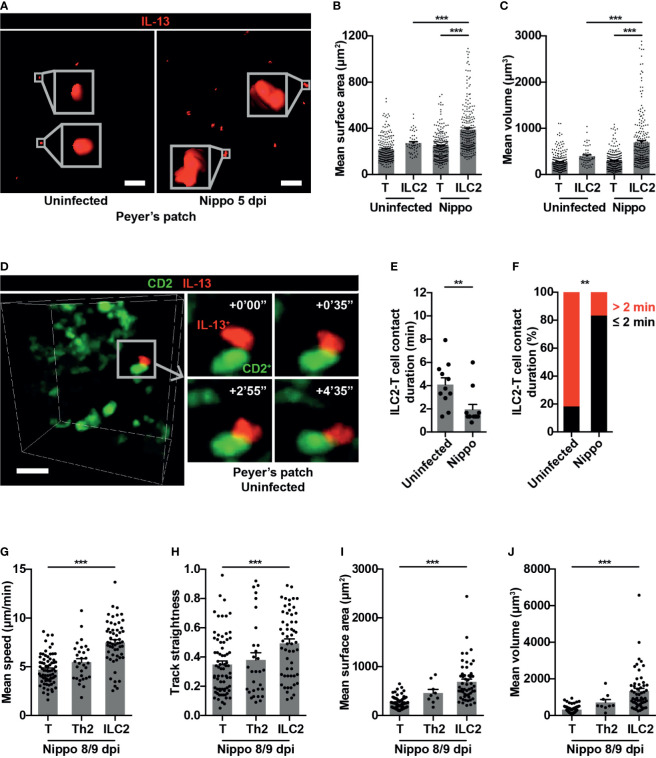
PP ILC2s show changes in size and cellular interactions following helminth infection. **(A)** Examples of cell size of PP ILC2s, uninfected and 5 dpi; scale bar 50 µm, Z 48 µm. **(B, C)** Quantification of **(B)** mean surface area and **(C)** mean volume of T cells and ILC2s, uninfected and 5 dpi; ***p < 0.001. Pooled data from 6-9 movies from 3 mice per condition, each dot representing one cell track. **(D)** Intravital example of ILC2-T cell interaction in uninfected PP; scale bar 20 µm, Z 48 µm. **(E)** Quantification of ILC2-T cell contact duration (min), each dot representing one interaction; **p < 0.01. **(F)** Quantification of proportions of ILC2-T cell contacts > 2 or ≤ 2 minutes; **p < 0.01 by Fisher’s test. Pooled data from 6-7 movies from 3 mice per condition. **(G–J)** Quantification of **(G)** mean speed, **(H)** track straightness, **(I)** mean surface area and **(J)** mean volume of CD2^+^ T cells, CD2^+^IL-13^+^ Th2 cells and IL-13^+^ ILC2s, 8/9 dpi; ***p < 0.001 ILC2 *vs* T. Pooled data from 3 movies, each dot representing one cell track.

To address whether the dynamic behaviour of these expanded ILC2s was altered following *N. brasiliensis* infection, we performed intravital imaging of PP in CD2-GFP/IL-13-tdTomato reporter mice. Overall, we found that IL-13^+^ ILC2s moved over greater distances post-infection (**Figures 2A–C** and **Movie S1**). In uninfected PP, ILC2s and T cells moved at similar speeds and with a similar level of directional movement, as evidenced by track straightness (**Figures 2D, E**). Following *N. brasiliensis* infection, T cell speed was unchanged, but directionality of movement was increased (**Figures 2D, E**). In contrast, PP ILC2s in *N. brasiliensis*-infected mice moved at significantly higher speeds than ILC2s in uninfected mice (mean 6.43 and 3.98 µm/min respectively; ***p < 0.001), and with increased directionality (**Figures 2D, E** and **Movie S1**). ILC2s also moved more quickly than T cells in the context of *N. brasiliensis* infection (mean 6.43 and 4.97 µm/min respectively; ***p < 0.001) (**Figures 2D, E**). We also observed an increase in ILC2 surface area and volume post-infection compared to ILC2s in uninfected PP, and compared to T cells post-infection (**Figures 3A–C** and **Movie S1**). No ILC2 or T cell division was observed during the course of intravital imaging of PP over a period of 13 hours in total.

Within T cell areas of the PP, ILC2-T cell interactions were only occasionally observed (**Figure 3D** and **Movie S2**) and were short in duration, both in homeostasis and post-helminth infection. Surprisingly, *N. brasiliensis* infection resulted in a reduction in duration of cellular interactions with T cells (mean 1.96 minutes *N. brasiliensis vs* 4.1 minutes uninfected; **p < 0.01) (**Figure 3E**), with a lower proportion of ILC2-T cell interactions lasting for more than 2 minutes post-infection (**Figure 3F**). Later (day 8/9) following *N. brasiliensis* infection, PP IL-13^+^ ILC2s but not CD2^+^IL-13^+^ Th2 cells showed similar increases in speed and directionality of movement as well as cell size, compared to CD2^+^ T cells (**Figures 3G–J**). No interactions between IL-13^+^ ILC2s and CD2^+^IL-13^+^ Th2 cells were observed at the later time points. Overall, these data reveal that intestinal *N. brasiliensis* infection leads to increased number, movement and cell size of PP ILC2s. Moreover, we observed shorter cellular contacts with T cells, suggesting that the antigen presentation function of ILC2s is not a prominent feature in gut-associated lymphoid tissues in the early response to helminth infection. Rather they are the dominant source of the type 2 cytokine IL-13, and show enhanced movement to potentially increase this cytokine’s distribution and range of action.

### ILC2s are Present in Popliteal LN and Show Dynamic Changes in Type 2 Inflammation

**Figure 4:**
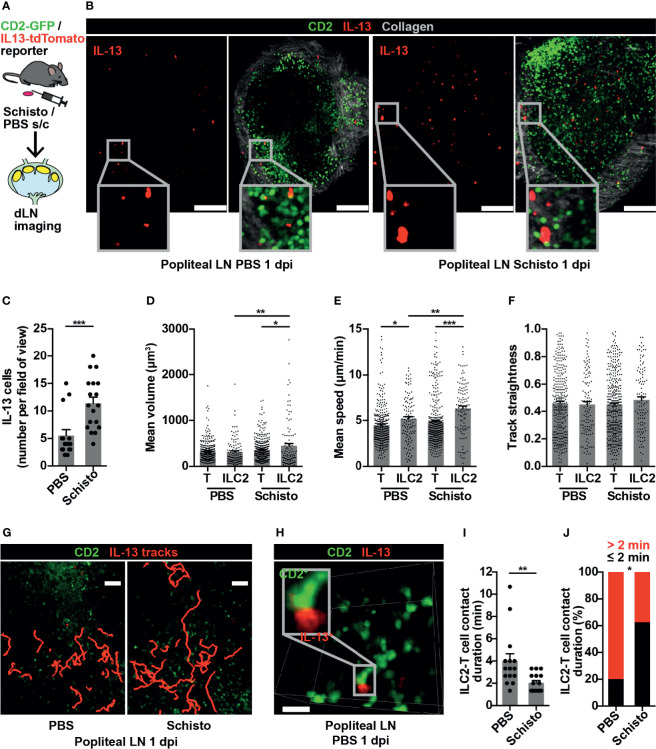
ILC2s are present in popliteal LN and show dynamic changes in type 2 inflammation. **(A)** Schematic of experimental setup. **(B)** Stitched images of popliteal LNs 1 day post immunisation (dpi) with PBS or *S. mansoni* (Schisto) eggs; scale bar 150 µm, Z 40 µm. **(C)** Quantification of ILC2 numbers per field of view; ***p < 0.001. Pooled data from 5-6 movies from 4 mice per condition, each dot representing one field of view. **(D–F)** Quantification of **(D)** mean cell volume, **(E)** mean speed and **(F)** track straightness of popliteal LN ILC2s and T cells 1 dpi; *p < 0.05, **p < 0.01, ***p < 0.001. Pooled data from 6 movies from 4 mice per condition, each dot representing one cell track. **(G)** Intravital image of ILC2 cell tracks in popliteal LN 1 dpi; scale bar 50 µm, Z 40 µm. **(H)** Intravital example of ILC2-T cell interaction in PBS-treated popliteal LN; scale bar 20 µm, Z 30 µm. **(I)** Quantification of ILC2-T cell contact duration (min), each dot representing one interaction; **p < 0.01. **(J)** Quantification of proportions of ILC2-T cell contacts > 2 or ≤ 2 minutes; *p < 0.05 by Fisher’s test. Pooled data from 6 movies from 4 mice per condition.

To confirm that these findings were not limited to ILC2s in gut-associated lymphoid tissues, we investigated ILC2 behaviour in a peripheral LN in the context of a type 2 immune response. Inactivated *S. mansoni* eggs, known to produce a strong Th2 response in mice (35), were administered into the hind hock, with PBS into the contralateral hock as a control, and the draining popliteal LNs imaged bilaterally at 1 day post-immunisation (dpi) (**Figure 4A**). IL-13^+^ cells were present in both PBS-treated and *S. mansoni*-stimulated popliteal LNs, and at this early time-point IL-13^+^ cells were exclusively ILC2s, with no IL-13^+^ T cells evident (**Figure 4B**). The number of popliteal LN ILC2s significantly increased, compared to control, following *S. mansoni* egg challenge (**Figure 4C**), and, as observed in PP post-helminth infection, *S. mansoni*-stimulated ILC2s had larger cell volumes compared to control ILC2s and to *S. mansoni*-stimulated T cells (**Figures 4B, D**). In addition, LN ILC2s moved at higher speed post-*S. mansoni* egg challenge compared with control LN ILC2s (6.36 and 5.18 µm/min respectively; **p < 0.01). In both control and *S. mansoni*-stimulated LNs, ILC2s also moved at a higher speed than T cells (**Figure 4E**). There was no statistically significant difference in ILC2 movement directionality post-*S. mansoni* egg challenge (**Figure 4F**), although some ILC2s moved over prolonged distances (**Figure 4G** and **Movie S3**). As observed in PP, brief ILC2-T cell interactions were also present in the popliteal LN (**Figure 4H** and **Movie S4**), and *S. mansoni* egg challenge resulted in a shorter duration of interactions (mean 2.04 minutes *S. mansoni vs* 4 minutes PBS; **p < 0.01) (**Figure 4I**) and a lower proportion of interactions lasting for more than 2 minutes (**Figure 4J**). Interestingly, 30-50% of IL-13^+^ ILC2s in lymphoid tissues expressed MHCII (**Figure S3**), with the potential for antigen presentation, but interactions with T cells at baseline were short-lived. Overall, these data generated from a peripheral LN mirror our observations in gut-associated lymphoid tissue, and support the conclusion that in the context of a Th2-inducing stimulus, ILC2s in lymphoid tissues act as early producers of type 2 cytokines and show increased cell movement, with the potential to enhance the spatial distribution of these cytokines, whilst undertaking fewer interactions with T cells.

### ILC2s Interact With T Cells in Small Bowel Following Helminth Infection

**Figure 5:**
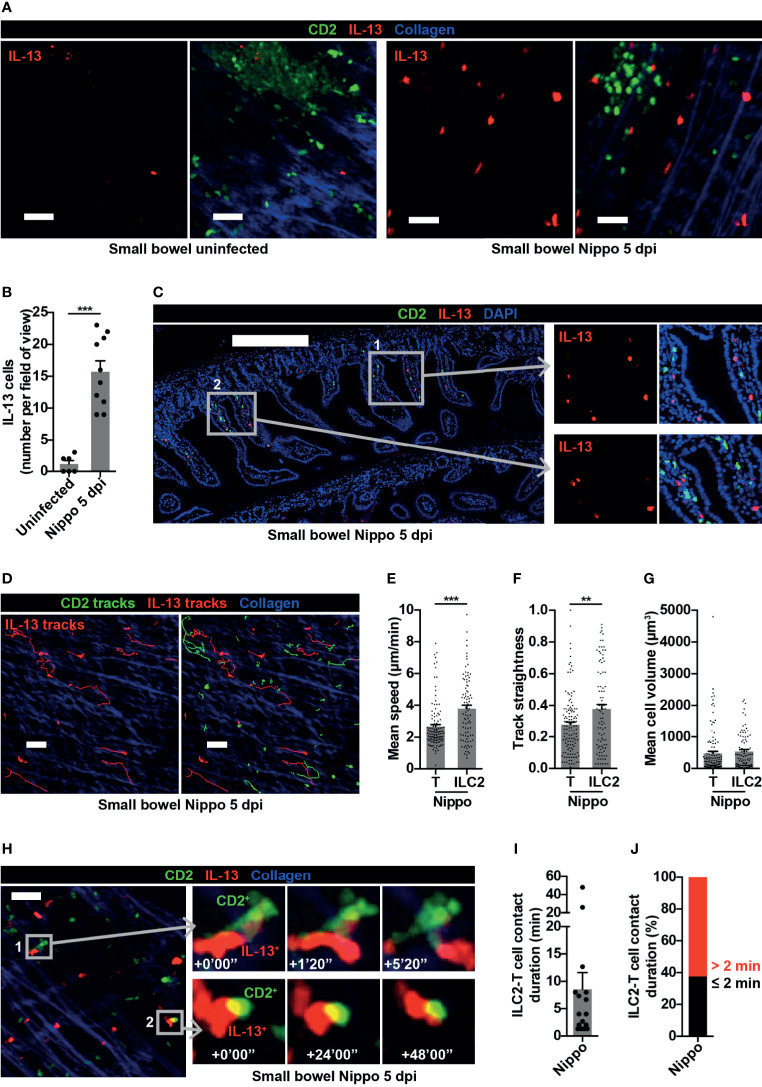
Small bowel ILC2s show altered movement and interact with T cells following helminth infection. **(A)** Intravital images of small bowel ILC2s, uninfected and Nippo 5 dpi; scale bar 50 µm, Z 16-30 µm. **(B)** Quantification of ILC2 numbers per field of view; ***p < 0.001. Pooled data from 4 movies from 1-3 mice per condition, each dot representing one field of view. **(C)** Stitched image of small bowel 5 dpi showing location of CD2^+^ and IL-13^+^ cells; scale bar 300 µm, Z 2 µm. **(D)** Intravital image showing ILC2 and T cell tracks in small bowel 5 dpi; scale bar 50 µm, Z 30 µm. **(E–G)** Quantification of **(E)** mean speed, **(F)** track straightness and **(G)** mean cell volume of small bowel ILC2s and T cells 5 dpi; **p < 0.01, ***p < 0.001. Pooled data from 3 movies, each dot representing one cell track. **(H)** Intravital examples of ILC2-T cell interaction in small bowel 5 dpi; scale bar 50 µm, Z 26 µm. **(I, J)** Quantification of **(I)** ILC2-T cell contact duration (min) with each dot representing one interaction, and **(J)** proportions of ILC2-T cell contacts > 2 or ≤ 2 minutes in small bowel 5 dpi. Pooled data from 3 movies.

ILCs are known to be important in shaping and generating immune responses at mucosal surfaces. We were therefore interested in examining the dynamic behaviour of ILC2s within the small bowel mucosa following *N. brasiliensis* infection. Whilst few IL-13^+^ ILC2s were present in uninfected mice at baseline, the number of IL-13^+^ ILC2s significantly increased 5 dpi (**Figures 5A, B**). These ILC2s were predominantly located in the mucosa in close proximity to T cells (**Figure 5C**). As observed in the PP at this time-point, all IL-13^+^ cells in the small bowel were ILC2s not Th2 cells (**Figure 5A**). ILC2s moved within the helminth-infected small bowel wall (**Figure 5D**, **Movie S5**) at higher speed (mean 3.79 µm/min ILC2s *vs* 2.65 µm/min T cells; ***p < 0.001) (**Figure 5E**) and directionality (**Figure 5F**) compared to T cells, with no difference in cell size (**Figure 5G**). Remarkably, in contrast to ILC2s in PP, ILC2s in the intestinal mucosa sustained longer interactions with T cells during *N. brasiliensis* infection [mean contact duration 8.54 minutes (**Figures 5H, I**)], with the majority of ILC2-T cell interactions lasting for more than 2 minutes (**Figure 5J**) and some very prolonged interactions (up to 48 minutes) evident (**Figure 5H**, **Figure S4A** and **Movie S6**). There was a trend towards a negative correlation between ILC2 mean migration speed and ILC2-T cell interaction duration, but this did not reach statistical significance (**Figure S4B**). These data demonstrated that, following helminth infection, small bowel mucosal ILC2s increased in number and displayed enhanced motility, potentially increasing the reach of the cytokines they produce and the likelihood of encountering other immune cells. They also undergo prolonged interactions with T cells, some of which were of a duration consistent with that required for antigen presentation. Altogether, our data suggest that ILC2s may have differing roles in mucosal tissues compared with lymphoid organs early in the course of a helminth infection.

## Discussion

Using two helminth experimental models, we have demonstrated that at early time-points following exposure to type 2 inflammatory stimuli, ILC2s increased in number and were the main source of IL-13 in both gut-associated lymphoid tissues and peripheral lymph nodes, as well as within the gut mucosa itself. In lymphoid tissues (PP and popliteal LN), IL-13^+^ ILC2s were present at baseline, and in response to type 2 stimuli ILC2s showed enhanced movement and increased cell size but reduced interactions with T cells despite their co-location, suggestive that their role is mainly to distribute type 2 cytokines. Although stimulated ILC2s in mucosal tissues (small bowel) showed enhanced movement, cell size was unchanged, and prolonged ILC2-T cell interactions were observed, including those of a duration compatible with immunogenic antigen presentation. This suggests that ILC2s may have distinct roles in lymphoid tissues compared with mucosal tissues. Surprisingly, it is at this latter site that contact-based T cell activation may occur. There may be tissue-specific differences in other immune cells, such as T cell production of IL-2 (36) and other cytokines, which influence local ILC2 dynamic behaviour.

Previous studies have used IL-13 reporter mice to identify ILC2s (3, 33). The use of CD2/IL-13 double reporter mice in our study allowed the important *in vivo* distinction between ILC2s and Th2 cells. A potential limitation is that not all ILC2s are IL-13^+^, and there may be differences in the dynamic behaviour between IL-13^+^ ILC2s and ILC2s as a whole. We showed higher IL-13 expression in gut-associated ILC2s compared to peripheral LN ILC2s, although our analysis used ST2 as a defining marker, which may only be expressed on a small subset of intestinal ILC2s (37). Alternative reporter mice utilised by other investigators to identify ILC2s may be useful for future studies of the dynamic behaviour of ILC2s. These include an IL-5 reporter, with tdTomato and Cre recombinase linked to the *Il5* gene, which has been used to fate map ILC2s. Here a high proportion of ILC2s express IL-5 in lungs and adipose tissue at baseline, although a lower proportion of ILC2s express IL-5 in LNs (22, 38). Alternatively, a KLRG-1 reporter has been used to visualise subsets of inflammatory ILC2s, although naïve ILC2s only show intermediate KLRG-1 expression (10, 39).

Studies in mice and zebrafish showed that immune cells responded to cytokines when within about 100 µm of the source but not beyond (40, 41), suggesting that cytokines have a limited area of influence in tissues. We found that helminth-stimulation increased ILC2 movement, some with prolonged cell tracks, with the potential to distribute cytokines over a wider area in lymphoid tissues, facilitating subsequent Th2 cell activation. This may contribute to the expansion of Th2 cells observed; No IL-13-producing Th2s were present at baseline, but we found an expansion at later time-points.

Following helminth stimulation, LN ILC2s showed reduced cellular contact. By contrast, in the small bowel mucosa we observed that some ILC2s had prolonged contacts with T cells, suggesting additional roles such as antigen presentation in mucosal tissues. A previous study showed that IL-33 stimulation led to expression of the co-stimulatory molecule OX40L on lung but not mediastinal LN ILC2s (19). Other studies have shown ILC2s to express MHCII (3, 17) and co-stimulatory molecules such as ICOS (19, 42), and contribute to T cell activation (4, 43, 44). To date there are only few intravital studies on the dynamic behaviour of ILC2s (32, 33) and ILC3s (45), and no data on their *in vivo* behaviour in LNs. Studies of DC-T cell interactions in LNs have demonstrated three different stages, with initial short contacts of 3-4 minutes, followed by prolonged contacts in stage 2 where peak contact time (64% over 60 minutes) occurred around 12 hours after initial encounter, but by 20 hours the majority of contacts had returned to short duration (26). Th2-polarising adjuvants were associated with shorter contact durations compared to Th1-polarising adjuvants (25 and 50 minutes at 12 hours, respectively) (29). Our ILC2-T cell contact durations in lymphoid tissues were similar to those at stages 1 and 3 at baseline, and shorter following helminth stimulation; in the gut mucosa, some prolonged interactions were observed, up to 48 minutes, consistent with some stage 2 interactions. Our experimental design did not allow us to specifically assess whether, similar to DC-T cell interactions, ILC2-T cell interactions occur in stages with different contact durations. Further studies are required to investigate whether the nature of these interactions changes at later time points following stimulation, for example whether interactions are more prolonged enabling antigen presentation to occur. However, we do provide the first dynamic imaging evidence of potential contact-dependent activation of T cells by ILC2s within the intestinal mucosa.

To date there have only been a few intravital studies of the dynamic behaviour of ILC2s (32, 33) and ILC3s (45), and no data on their *in vivo* behaviour in LNs, PPs or intestinal mucosa. Our results therefore provide novel insights into ILC2 dynamic behaviour, although the underlying molecular mechanism governing the increased movement observed post-challenge requires further investigation. IL-13^+^ ILC2s were not evident within the small intestine at baseline, precluding investigation of their motility at steady state within gut mucosa. Therefore, further studies using alternative reporters will be required to determine whether our observations of altered ILC2 motility and T cell interactions in infection compared to homeostasis in secondary lymphoid organs hold true for ILC2s within mucosal tissues. We showed migratory speeds of helminth-stimulated ILC2s of 6.4 µm/min in PP and popliteal LN, and 3.8 µm/min in small bowel mucosa. A previous study using sectioned lung explants from IL-33-stimulated mice showed ILC2 speed around 3 µm/min, with movement reduced by CCR8 blockade (33), whereas another study showed dermal ILC2 speed to be slower at around 2 µm/min at baseline (32). Our data are likely to better reflect true *in vivo* speeds due to the challenges of maintaining tissues in explant studies.

In summary, our study provides the first dynamic imaging of ILC2 *in vivo* in the intestine and PP. We show that IL-13^+^ ILC2s increase their speed and range of movement following helminth infection in both tissues, but that prolonged T cell contacts are only observed within the mucosa and not within lymphoid tissues. Our findings have important implications for our understanding of ILC2 biology and the way in which they facilitate adaptive immune responses.

## Data Availability Statement

The raw data supporting the conclusions of this article will be made available by the authors, without undue reservation.

## Ethics Statement

Animal studies were reviewed and approved by the Laboratory of Molecular Biology Animal Research Ethics Committee in accordance with The Animals (Scientific Procedures) Act 1986 UK.

## Author Contributions

LL, JW, HJ, and SS performed experiments. LL, MI, PF, AM, and MC analysed and interpreted data. AM and MC conceived the project. LL and MC wrote the first draft of the manuscript. All authors contributed to the article and approved the submitted version.

## Funding

This study was supported by grants from the UK Medical Research Council (U105178805) and Wellcome Trust (100963/Z/13/Z, 104384/Z/14/Z).

## Conflict of Interest

The authors declare that the research was conducted in the absence of any commercial or financial relationships that could be construed as a potential conflict of interest.

## Publisher’s Note

All claims expressed in this article are solely those of the authors and do not necessarily represent those of their affiliated organizations, or those of the publisher, the editors and the reviewers. Any product that may be evaluated in this article, or claim that may be made by its manufacturer, is not guaranteed or endorsed by the publisher.
