# Supplementary material for: Group 2 Innate Lymphoid Cells Exhibit Tissue-Specific Dynamic Behaviour During Type 2 Immune Responses
Source: Front Immunol. 2021 Aug 17;12:711907. doi: 10.3389/fimmu.2021.711907 (PMC8415880; doi:10.3389/fimmu.2021.711907)
Supplement: Supplementary file 1 [file DataSheet_1.pdf]

Supplementary figures

Figure S1

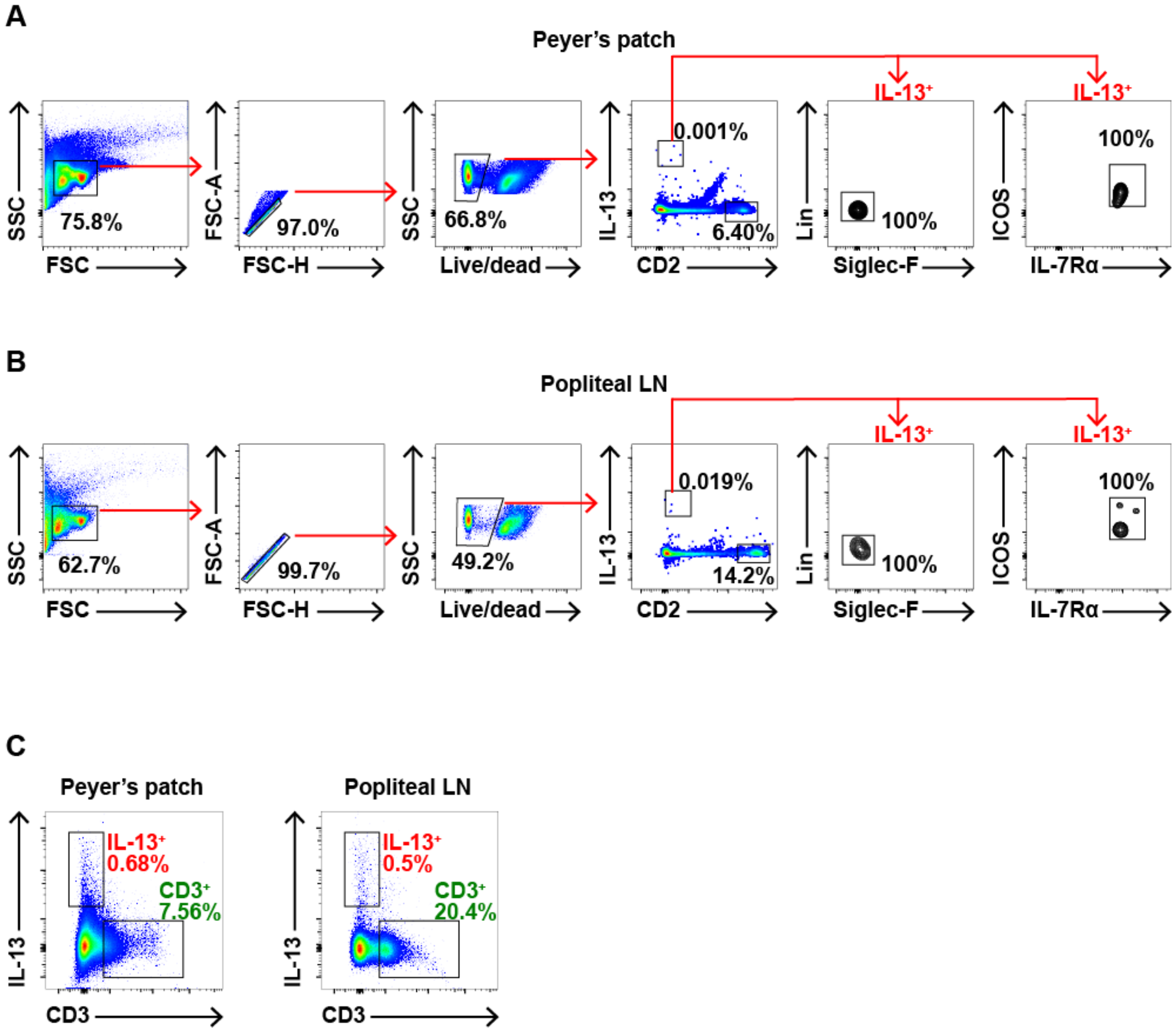

Figure S1. Specificity of IL-13^{+} cells for ILC2s. (A-B) Flow cytometry plots showing proportions of ILC2s (Lin^{neg}Siglec-F^{neg}IL-7Rα^{+}ICOS^{+}) in CD2^{neg}IL-13^{+} cell populations in (A) PP and (B) popliteal LN. (C) Flow cytometry plots showing proportions of IL-13^{+} cells and CD3^{+} cells, gated on live cells, in PP and popliteal LN.

Figure S2

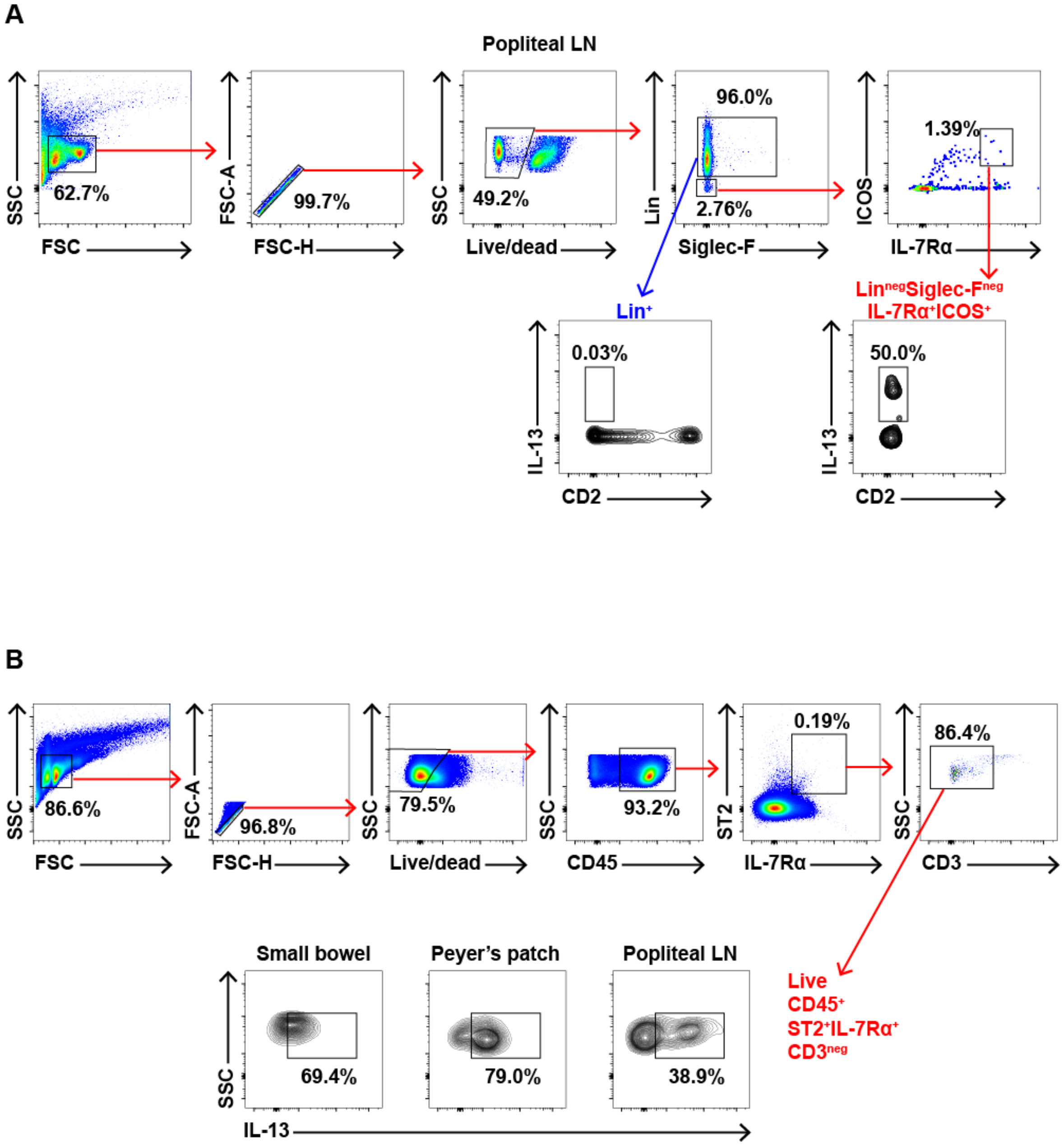

Figure S2. IL-13 expression by ILC2s. (A) Flow cytometry plots showing proportions of IL-13-expressing cells in ILC2 and lineage-positive cell populations. (B) Flow cytometry plots showing proportions of IL-13-expressing ILC2s ($CD45^{+}ST2^{+}IL\text{-}7R\alpha^{+}CD3^{neg}$) in mucosal and lymphoid tissues.

Figure S3

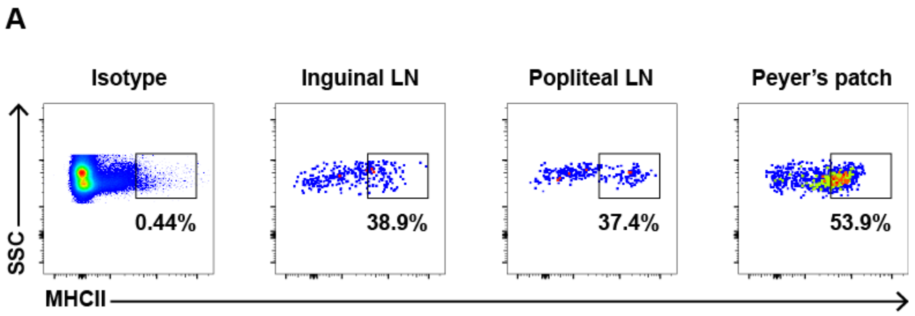

Figure S3. MHCII expression by ILC2s. (A) Flow cytometry plots showing expression of MHCII by ILC2s (ST2^{+}IL-7Rα^{+}CD3^{neg}IL-13^{+}) in lymphoid organs at baseline.

Figure S4

A

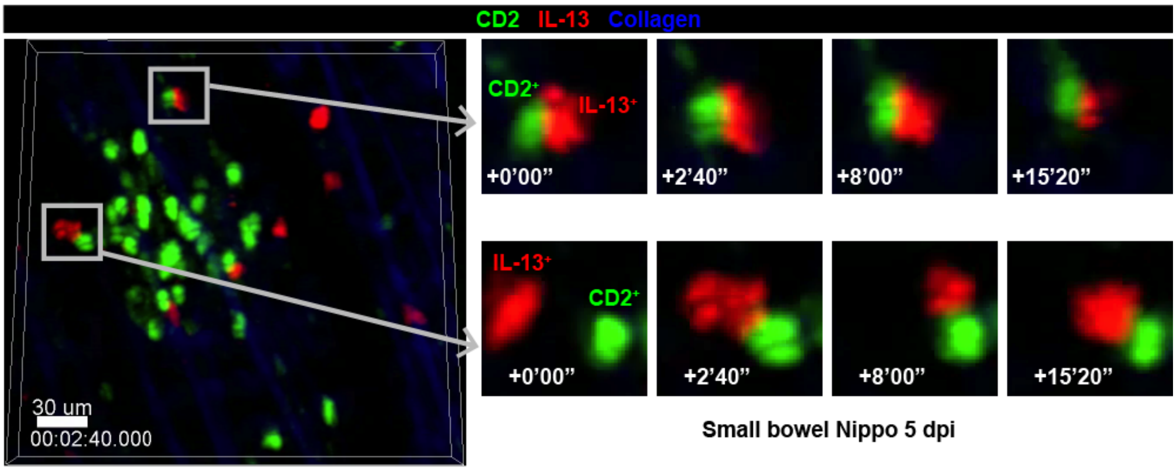

B

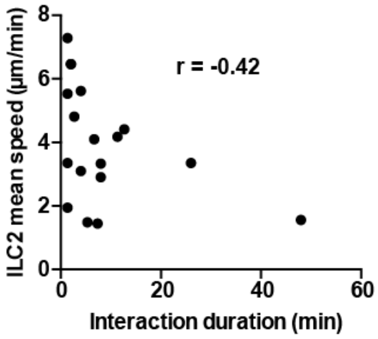

Figure S4. ILC2s interact with T cells in small bowel mucosa following helminth infection. (A) Intravital examples of interactions between IL-13^+ ILC2s and $CD2^+$ T cells in the small bowel mucosa, Nippo 5 dpi; scale bar 30 µm, Z 16 µm. (B) Graph showing relationship between ILC2 mean migration speed and ILC2-T cell interaction duration, Spearman correlation r = -0.42, p = 0.08.

Supplementary movies

Movie S1. PP ILC2s show altered size and movement following helminth infection. Intravital movies of ILC2s, without and with cell tracks, in PP uninfected and *N. brasiliensis* (5 dpi); scale bar 50 μm, Z 48 μm, movies at 10 fps.

Movie S2. ILC2s occasionally and briefly interact with T cells in PP at baseline. Intravital example of ILC2-T cell interaction in uninfected PP; scale bar 20 μm, Z 48 μm, movie at 3 fps.

Movie S3. Popliteal LN ILC2s show dynamic changes following type 2 inflammation. Intravital movies of ILC2s with cell tracks in popliteal LN following PBS treatment or *S. mansoni* egg challenge (1 dpi); scale bar 50 μm, Z 40 μm, movies at 10 fps.

Movie S4. ILC2s occasionally and briefly interact with T cells in popliteal LN at baseline. Intravital example of ILC2-T cell interaction in PBS-treated popliteal LN; scale bar 20 μm, Z 30 μm, movie at 3 fps.

Movie S5. Small bowel ILC2s show dynamic changes following helminth infection. Intravital movie of ILC2s, without and with cell tracks, in small bowel mucosa following *N. brasiliensis* infection (5 dpi); scale bar 50 μm, Z 30 μm, movie at 10 fps.

Movie S6. ILC2s interact with T cells in small bowel mucosa following helminth infection. Intravital examples of ILC2-T cell interactions in small bowel mucosa following *N. brasiliensis* infection (5 dpi); scale bar 30 μm, Z 26 μm, movie at 5 fps.
